# Chronic challenges: picturing chronic disease by the World Health Organization

**DOI:** 10.1136/medhum-2023-012737

**Published:** 2024-08-07

**Authors:** Alexander Medcalf, Karl Atkin

**Affiliations:** 1 History, University of York, York, UK; 2 Sociology, University of York, York, UK

**Keywords:** history, popular media, medical humanities

## Abstract

Chronic diseases are among the leading causes of mortality in the world, the subject of major regional and international efforts to tackle shared risk factors, implement prevention and control measures and set national targets as part of the drive towards universal health coverage. Yet there is a growing conviction that chronic diseases suffer an image problem. It has been suggested that the terminology ‘dulls the senses' to the problems, and in an age where the mass media affords unprecedented opportunities to inform and persuade people to care about their health and that of others, chronic disease representation remains a contested and much debated issue.

This article investigates how WHO created and disseminated visual narratives to raise popular consciousness and build a visual vocabulary around chronic disease in the second half of the 20th century. It examines the measures taken to conceptualise, photograph and publicise chronic diseases, and considers who had control over their representation. In focussing predominantly on cancer, diabetes and cardiovascular disease, it reveals different narratives; the power of scientific and technological progress; individual and community action for health; promising utopian and parallel dystopian visions. It embeds these in a production context which reveals an intricate picturing process involving overcoming challenges of representation. It uses this historical background to discuss issues relating to how chronic disease and chronic pain have been narrated visually, such as the ideas of emotional response, moral failure, how people navigate the ‘risk society’ and ultimately the concerns regarding the intentional and unintentional influence that the media can have on the image of disease given to society.

## Introduction

 While public attention to disease has expanded over time, different conditions have captured that attention to varying degrees (Armstrong, Carpenter, and Hojnacki 2006, 731). Non-communicable diseases (NCDs), also known as chronic diseases, are said to suffer from an image problem. It has been suggested that the terminology ‘dulls the senses to the urgency of the problems involved’ (Aluwihare 2002, 173; Allen and Feigl 2017, 129–130), and at a time when media technologies offer unprecedented opportunities to communicate with people about health topics, effective chronic disease representation remains a much-debated issue. The difficulty in stimulating media interest in chronic conditions is said to stem from many factors: the physical suffering can be all but invisible to the naked eye; each person will face different limitations as a result of their condition (Monroe 2017, 17) and there is the question of how to sensitively yet convincingly visualise the personal, emotional impact of chronic conditions on individuals, families and communities. The challenge of how to capture those who live well with chronic conditions only adds to this complexity.

Maher and Sridhar have examined why certain ways of framing disease resonate with politicians and the public while others do not. One conclusion is that chronic conditions commonly lack emotion and a ‘human face’ (Maher and Sridhar 2012, 1–10). To compete for attention effectively, it is assumed that they require an emotive ‘sufferer’. This is borne out in advice given to those involved in representation: a 2006 WHO advocacy guide, for example, recommended that photographs focus on people (‘at the end of the day chronic diseases affect individuals’); that they should engage with these individuals (‘close up and eye contact work best’) and that they should appeal to people’s emotions to ‘stand out from the clutter and drive an emotional response’ (WHO 2006, 33). Scholars argue that the stories we choose to tell, as well as those we are exposed to in the course of everyday life, play an important role in experiencing illness. In *The Wounded Storyteller*, Arthur Frank argues that telling the story of illness is moulded by rhetorical expectations that the storyteller has internalised from various places, such as hearing a relative describe an illness, seeing television commercials for non-prescription remedies or working out what ‘counted’ as the story that a doctor wanted to hear. Frank argues that people become storytellers who ‘have learnt formal structures of narrative, conventional metaphors and imagery, and standards of what is and is not appropriate to tell’ (Frank 2013, 3). There has been a recent flourish of scholarship examining the ways in which chronic disease and chronic pain have been narrated (van Hout, van Rooden, and Slatman 2022), which builds on competitions, web-based initiatives and projects such as Capturing Chronic Illness which explore how chronic illness can be articulated visually, and ask ‘what does the visual offer when words fail?’^1^

But what is often absent from such discussions is detailed historical inquiry into the evolution of attempts to build interest in, and narratives around, chronic conditions. We require more information about how a visual vocabulary of chronic disease has been established over time: what has been brought to the fore or omitted; how and what have people been encouraged to see and feel and what factors have informed the design and procurement of imagery. Such inquiry can be used to build a clearer picture of the sorts of dominant narratives over time, as well as encouraging debate about why they have become so entrenched.

This article contends that the history of chronic disease photojournalism can be used to explore how people have been persuaded to care about the chronic disease issue as well as the impact of this influence. To do this, it analyses the photojournalism of WHO between 1948 and 1990. Serlin (2010, xiii) argues that health issues are ‘unknowable’ without visual representations, and that visual media plays an important role in establishing a frame of understanding. Throughout much of the 20th century, photography played a key role within this. More than just a way of recording objectively, the photograph used as part of a public information campaign is laden with evidence about what the individual or organisation responsible for its production wanted to communicate, what they thought about potential audiences and how they hoped the image would be interpreted. As Sontag (1990, 6–7) argued, ‘photographs are as much an interpretation of the world as paintings and drawings are’. While there are always multiple ways in which an image can be understood, equally there is usually a favoured or dominant reading, a broad acceptance of the image’s message that those who commissioned the work anticipate most people will take. The analysis throughout this article is based on the reasonable assumption that organisations such as WHO constructed photo campaigns with this dominant reading in mind. This is captured in the difference between ‘vision’, what the human eye is physiologically capable of seeing and ‘visuality’, how vision is constructed in various ways and how we are made to see (Foster 1988, ix). This does not mean that all viewers accepted or comprehended the meaning in the same way, but that they would nevertheless have been exposed to certain organisationally endorsed ways of seeing.

While historians have primarily focused on WHO’s policies and technical leadership in tackling health problems, its senior management have long acknowledged that the Organization’s work depends on public understanding and support. They quickly turned to photography, believing that it could help to foster attention and understanding across geographical, cultural and linguistic barriers. Using imagery supplied by prominent photojournalists and celebrated agencies such as Magnum, by the 1960s WHO had assembled an extensive collection of photographs capable of illustrating health activities and achievements in almost any area of the globe (WHO 1968, 293–94). These were given pride of place in WHO’s own public-facing publications, the *WHO Newsletter* (1948–1958) and its successor *World Health* (1958–1998), which emulated the style of mass market photo magazines like *Life* and *Look* (David and Rodogno 2015, 225–26). Furthermore, WHO was successful in featuring *World Health’s* photo stories in popular and well-regarded periodicals (Medcalf 2018, 115–17), meaning that its photojournalism had a wide reach among health policymakers and the general public.

Photo stories enabled WHO to give potentially opaque technical assistance work a human drama, as well as providing apparent evidence of progress and success. While it may have been visually arresting, WHO’s photojournalism cultivated very specific definitions of what it was to be healthy, to suffer disease and to engage with the medical establishment. Although hard-hitting photographs helped to garner attention, they also carried the potential for unintended and unforeseen consequences. Photographs used as part of health advocacy work can, for instance, encourage a false sense of hope by promising treatments or the possibility of cure which may not be available to all. Similarly, a focus on solutions can reduce people’s sense of risk. Used repetitively, formerly successful photo stories can lead to compassion fatigue over time, dampening people’s concern for an issue. This article considers how WHO’s images of chronic disease were intended to work, what informed their design as well as how this changed over time. WHO expended significant resources on researching and procuring meaningful imagery: its archives hold material for 48 photographic missions on cancer, cardiovascular diseases and diabetes between 1959 and 1977 (photographic missions continued after this point but only the published material survives). The sections which follow use the products of these missions; unpublished negatives, published photo stories and reports produced by the WHO Division of Public Information. The photographs’ symbolic content, the influences acting on their construction and the media in which they were presented are used to understand how the process of vision was mediated and consider the consequences of this.

Not all chronic diseases will be covered by this article, which will focus on cancer, cardiovascular diseases and diabetes given that WHO viewed these as the most high-profile global challenges, and continues to do so in more recent times (WHO 2013). Similarly, we do not propose an exhaustive history of chronic diseases at WHO. While this article contributes to clarifying this picture, along with the work by Weisz and Vignola‐Gagné (2015), a detailed history of WHO’s chronic disease departments remains to be written. The intention here is rather to unpack the myriad ways of representing conditions which together have been described variously as constituting an ‘invisible’ epidemic.

### Coming into vision: chronic disease at WHO

As significant as they may have been to those suffering their effects, longstanding chronic diseases were not well-understood in the 19th century and were therefore not a major category for public health activities (Weisz 2014, 5). But as public health initiatives began to reduce mortality from infectious diseases, and as people lived longer as a result (Tuchman 2009, 1140–41), the concern turned to what conditions people ‘lived with’ rather than what they ‘died from’ (Porter 1999, 298). Cancer, diabetes and cardiovascular diseases began to attract greater attention throughout the first half of the 20th century, being viewed as part of a swathe of harmful aspects of modern, urban living including pollution, accidents, poverty, overcrowding and poor housing (Weisz 2014, 17). Yet, because the aetiology of these diseases remained imperfectly understood, it was harder to communicate with the public about them. Although various methods were used to get the message across, these differed by region and organisation. For instance, while the American Society for the Control of Cancer developed a range of public-education programmes (Cantor 2007a, 40–41), underdeveloped cancer awareness efforts in Britain reflected the fear that such attempts would induce either excessive fear or undue optimism about the possibility of a cure (Cantor 2007b, 9; Barnes 2008, 10).

Following its inauguration in 1948, WHO’s immediate priorities included prominent communicable diseases such as malaria, tuberculosis and yaws. But chronic conditions were acknowledged as an intractable issue meriting further attention. In his annual report of 1952, WHO’s inaugural Director General Brock Chisholm noted that while numerous communicable diseases had been brought under control in many high-income countries, chronic diseases and the so-called diseases of old age had simultaneously escalated (World Health Organization and Chisholm 1953, 4). Subsequent reports of the 1950s and early 1960s demonstrate a growing interest, yet despite incremental advances in science and healthcare, WHO recognised that tackling chronic disease necessitated overcoming somewhat unique challenges.

In Amsterdam in 1957, a flagship WHO symposium on ‘Public Health Aspects of Chronic Disease’ concluded that chronic diseases were very difficult to approach and solve because the symptoms were so various, the aetiology and pathology multiple and obscure, and the onset usually insidious. Correcting habits through health education was seen as important but far from straightforward because changing habits meant altering deeply rooted feelings and practices. Emotional sentiments weighed strongly against arguments to reason; perceived ‘faulty’ dietary habits were hard to correct because food meant more to people than simply the intake of calories (Regional Office for Europe of the World Health Organization 1958, 33–35). Moreover, the consequences might seem too distant or remote; the idea of chance and risk played a significant part in how warnings were received. But ultimately, the problems associated with chronic diseases were seen to lack the immediacy and urgency that characterised communicable diseases.

It was also harder to attract interest, funding and expertise to these conditions. A 1958 committee called attention to the lack of experienced epidemiologists for the study of chronic diseases and suggested that WHO urgently promote a broad programme of training to meet this deficiency (World Health Organization and Candau 1959, 17–18). Chronic conditions suffered from a reputation as diseases predominantly of advanced age: it was not easy to attract the interest of medical students and practitioners, partly because in the past, patients in chronic wards of hospitals had been regarded as inferior and ‘lacking dramatic excitement’ (official records of World Health Organization 1959, 62). In contrast, communicable diseases had a certain glamour, arguably augmented by WHO’s own photojournalism, which commonly focused on miraculous ‘before and after narratives’ based on photographs showing patients stuck down by disease followed by ones depicting a full return to health. As the analysis by David and Rodogno shows, a crucial ingredient within this was the representation of pain and suffering (David and Rodogno 2015, 224–32). Articles sought to and arouse readers’ emotions: photo stories drew on recognisable and understandable themes about development and progress, and used a cast of villains, victims and saviours (David and Rodogno 2015, 224). As part of this, the human face of communicable diseases was highly visible; miserable faces underscored the need to solve problems; damaged ones stressed the urgency and magnitude of problems and happy ones symbolised the resolution (Medcalf 2018, 80). The heightened joy and despair seen on close-up faces, often of children, was a key feature of early photo stories. It served to confirm textual descriptions of lives altered by disease but then restored, which Arthur Frank refers to as the ‘restitution narrative’—whereby people are returned to a ‘normal’ state of health—and the ‘modernist expectation that for every suffering there is a remedy’ (Frank 2013, 80). This focus on the individual was intended to make viewers feel for the plight of the ‘victim’ and pay attention, and to further educate them in a pervasive model of how illness should be understood and narrated. This met social expectations for restoring health while maintaining hope, which avoided the lack of plot or imagined end associated with more anxiety-provoking chaos narratives (Frank 2013).

In terms of their chronic illness photo stories, WHO’s media teams were faced with a number of questions. In the case of malaria, for instance, the mosquito was a recognisable and easily understandable villain. But who or what was the villain of chronic disease, and how should it be presented? Was it the invisible cancer, or the patient’s own habits? If the latter, could the patient be considered a victim in the same way as, for example, someone suffering from malaria, leprosy or sleeping sickness? Additionally, how could the internal effect of chronic disease on the body, its invisibility, be visualised? And, ultimately, how could WHO show what defeating such conditions looked like? WHO ultimately needed to communicate with people about chronic disease, to explain issues which were not readily understood, allay fears and report on progress. To address this in the climate of the 1950s and 1960s it relied on a very particular set of visual narratives which are examined below.

### Yielding to science

People were encouraged to view the post-1945 international health landscape with considerable optimism. A new era of scientific collaboration, new medicines and ‘weapons’ in the fight against disease all contributed to a renewed sense of confidence. Indeed, WHO’s second Director-General, Marcolino Candau, prophesied that within the foreseeable future yaws, syphilis, smallpox, tuberculosis, leprosy and finally cancer and heart disease would ‘yield to science’ (Cueto 2007, 60). WHO magazines *Newsletter* and then *World Health* regularly featured headlines brimming with anticipation, such as ‘WHO and the Advancement of Science’ (1950), ‘WHO and the Atom’ (1956), ‘Machines of Modern Medicine’ (1967) and ‘Space Medicine and Mankind’ (1969). They were accompanied by photographs which offered readers visual confirmation that they were living in an extraordinary age of modern science. On occasion, the addition of fanciful pen-and-ink illustrations invited readers to consider where technology might take humankind next.

Initially, chronic diseases were placed firmly into this overarching narrative of scientific progress. Although internal reports may have sounded more caution about the prospects of cure, the WHO’s public-facing periodicals presented developments in the field of chronic diseases as dramatic and boundary pushing. WHO appreciated that when, for example, cancer was usually mentioned in the public sphere, it was in connection with the death of a prominent public figure, such as a politician or film star. It therefore sought to revise the view that diagnosis was equivalent to a ‘death sentence’ (World Health 1960), believing that sensitive introduction to scientific and technical views could help change public perceptions. As such, articles and photo stories reported on a procession of new materials and machines, which extended the horizons of modern medicine. They showed plastics that repaired or replaced damaged organs, photoelectric cells which monitored heart activity and miniature cameras that peered inside the body. Each contributed to an optimistic narrative that emphasised control over chronic disease, including raising the possibility of cure. Little attempt was made as part of this to engage with the life of patients or the experience of living with disease. Their ‘story’ was downplayed against that of advancing technology and treatment.

In terms of setting, most of these photo stories focused on laboratory scenes and operating theatres filled with unfamiliar equipment and gleaming machinery. The multiple artificial light sources commonplace in these locations bounced and reflected off the apparatus serving to give a futuristic gleam and an aura of curiosity. One such article on cardiovascular disease, titled ‘Specialists working in a highly mechanised setting’, described a machine designed to take over the functions of a patient’s heart and lungs during an operation (World Health 1960). The accompanying photograph pictured a surgeon and assistant surrounded by an array of bright and complex machinery. Neither engaged the camera directly but were seen absorbedly scrutinising samples and readouts. One held up a glass beaker to the light in a familiar and well-rehearsed pose which the viewer was meant to interpret as the best minds and technologies being brought to bear on the problem. As Hansen (2009, 247) notes in the case of *Life* magazine’s visual content, an image which captured a proliferation of test tubes was intended to speak symbolically of the ‘endless experiments that led to discovery’. In another WHO photo story, ‘A Heart Seen by a Surgeon’, the author explained how the ‘man with the scalpel’ approached a difficult case, accompanied by photographs which showed a diseased heart and the ‘spare parts’ used to repair defects in the heart and blood vessels (World Health 1965). The photographs gave readers privileged visual access inside the human body and were intended to spark the reader’s interest with information and images which they were unlikely to have encountered before. But they also firmly suggested advanced technology as a saviour. The photographic narrative emphasised that humanity was increasingly reliant on progressively more advanced machinery, and that it was correct to do so: this was what ‘modern medicine’ looked like.

Patients, by contrast, were presented as largely powerless and passive individuals in the hands of this technology and the specialists who operated it. Patients lacked identity, in that observers learnt little of their lives other than to know their chances of recovery. Faces were obscured or shrouded in shadows. They were often anonymous and invariably presented alone. This absence of family or friends is peculiar given that the aforementioned Amsterdam symposium had emphasised the crucial role played by social groups in offering support to those living with chronic disease. Instead, patients were pictured on the operating table, in consultation with or being examined by medical professionals. In a photo story on the ‘ultra-modern facility in the radiotherapy room’, the photographer Paul Almasy captured a ‘patient’ (who was most likely a model drafted in to populate the scene) with eyes closed, under a glass cover, surrounded by medial apparatus. While it is impossible to unequivocally discern Almasy’s intentions, the photograph (Almasy 1969) appears to recall the famous scene from the 1937 film *Snow White and the Seven Dwarfs* in which the titular character lies in a glass coffin in a deathlike torpor. Perhaps WHO’s editors intended those familiar with the film to draw a parallel with this patient serenely waiting for the awakening ‘kiss’ of technology; such a reading would align with WHO’s avowed aim to procure attractive and popularly appealing content. Regardless of whether the viewer made this particular connection, they were served with a clear message of technology’s potential to save.

**Figure 1:**
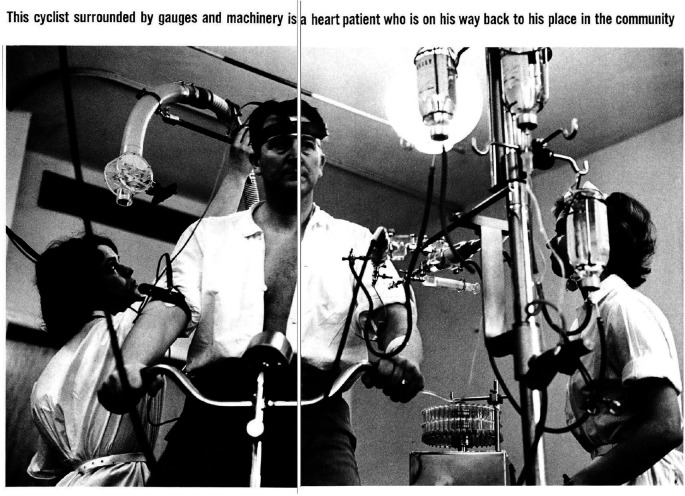
Photograph by WHO, Paul Almasy, of a cyclist surrounded by gauges and machinery from World Health, cardiovascular diseases special issue, 1965, pages 18–19. Copyright WHO/Paul Almasy, 1965. Figure permission obtained.

One 1965 special issue on cardiovascular disease featured a double-page photograph of a heart patient ‘riding’ a stationary bicycle as their breathing, pulse rate and heart function are observed. Surrounded by gauges and tubing, the patient was flanked by two nurses who monitored and adjusted the machinery (figure 1). The tubes, wires and straps protruding at multiple angles give the outwardly everyday activity a sense of investigation and calculation. The fact that the image was interpretable, but the specific actions less clear, emphasised a further degree of trust and the reliance on professionals. The unpublished variants on the accompanying contact sheet show how this narrative was constructed before a definitive image was arrived at, and also showcase many of the elements described above: the patient prone and shadowy; the doctor in a position of power; staff consulting and adjusting machinery and the dominance of equipment. Only one showed a more relaxed, conversational scene. The ultimately unused variants on the contact sheets suggest a rigorous process of procurement and selection, as well as indicating the kind of imagery sought from the outset.

**Figure 2:**
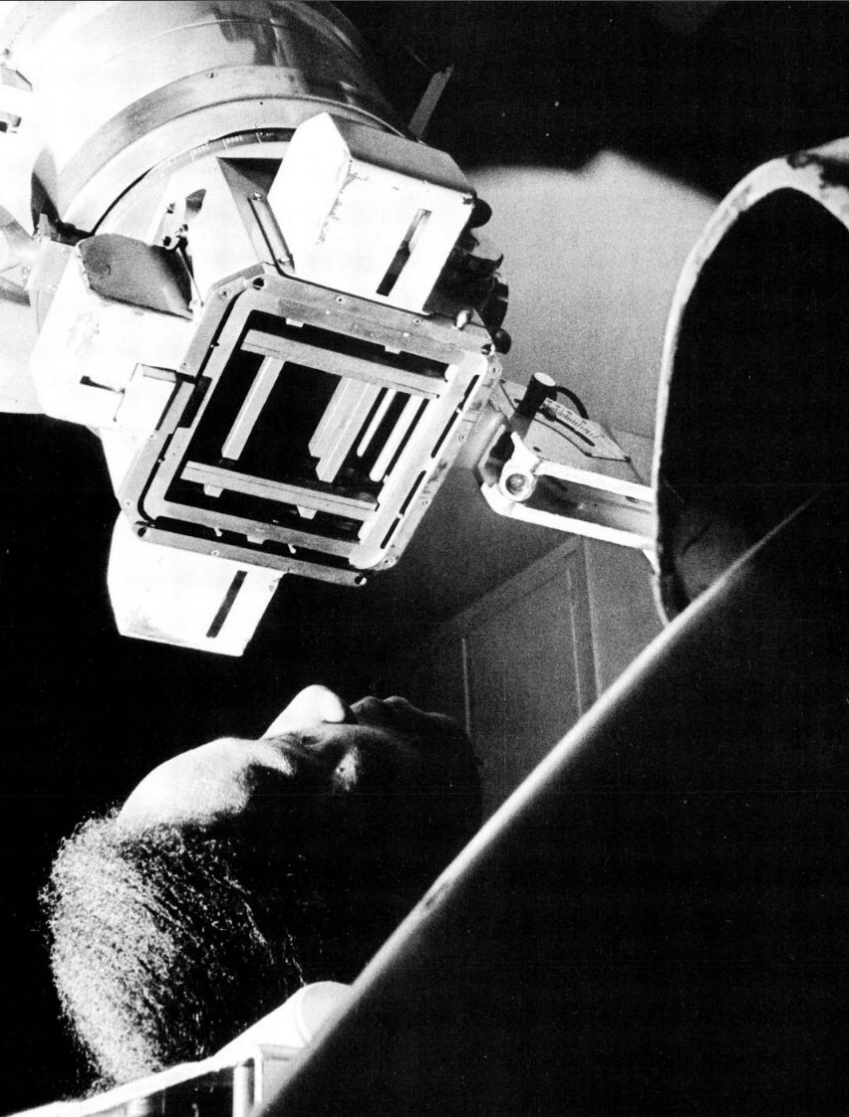
Photography by WHO, Eric Schwab, reproduced in World Health, Cancer special issue, September 1964, page 15. Copyright WHO/ Eric Schwab, 1964. Figure permission obtained.

This overarching focus on technology was also given a militaristic tone, with techniques and equipment portrayed as ‘weapons’ and ‘defence mechanisms’, suggesting that humanity was waging a war against conditions that had yet to yield to science. The cancer campaign, for instance, needed to be ‘vigorously waged on several fronts’ (World Health 1960). The inclusion of military symbolism was a tactic borrowed from earlier public health campaigns, given new life within the symbolic language of the Cold War period (Cueto 2007, 65) and worked with a parallel language of disease which focused on ‘invasion’, ‘chaos’ and colonisation (Sontag 1991, 64–66). With the chronic disease challenge established as a ‘war’, it was the task of photographers to show progress from the front lines, key battles, new weapons, territory gained and stress the efforts required for victory. In a 1964 *World Health* special issue on cancer, viewers were shown a partial view of a patient being irradiated by a ‘cobalt bomb’ (World Health, 1964) (figure 2). Eric Schwab’s arresting photograph placed the viewer in an unusual perspective underneath the machine looking up at the rear of the patient’s head as they lay prone. The alien-looking instrument looms over patient and viewer, these relative heights further suggesting the power of technology. While the framing of this particular photograph was novel and eye-catching, the article’s language recalled earlier reporting, such as *Life* magazine’s feature on a 200 000 V deep therapy X-ray machine and the Crocker Laboratory’s 1.24 million volt X-ray machine identified as the ‘Biggest Gun in the War Against Cancer’ (Hansen 2009, 215).

Such photo stories showed little active agency on the part of the patient. They did little to engage with the chronicity or impact of the diseases, or much about the life lived with conditions. This is surprising given that it was a common technique in the WHO publications by this time to use ‘before’ and ‘after’ photographs of patients cured of leprosy or smallpox, or the archetypal village freed from the scourge of malaria. But overall, WHO shared commonalities with other agencies at this time in the idealisation of patients as stoic and compliant individuals who responded to disease rationally and reasonably, and who followed medical advice (Godtschalk 2012, 13). On the other hand, neither did the organisation opt to regularly show pictures of people smoking, drinking or indulging in habits considered to be problematic. Although ‘faulty’ habits had been identified, WHO’s photo stories engaged in little blaming or stigmatising of behaviours likely to contribute to some chronic conditions.

Medical actors, by contrast, were presented as individual, resolute, heroic yet silent guardians; specialists for humanity to put its trust in. They were commonly staged in ways suggestive of the glamour of delivering humanity from the scourge of disease, but this was a mysterious and anonymous glamour. Although names were included and research described, WHO invariably continued a trend, started in the interwar years, whereby individual scientists were less likely to be known, and were presented as less accessible and differentiated in their personalities (Hansen 2009, 126). In a photograph of Professor Lars Werkö of the University of Gothenburg, Sweden, speaking with one of his patients (by Paul Almasy), a shadowy effect was produced, and a clear power dynamic referenced as patient and doctor ‘talk it over’ (World Health 1965). The doctor was physically taller, and his stature connoted power, confidence and knowledge. The presentation of scientists and the doctors by WHO was in contrast to the portraiture styles of historic ‘heroes’ of medicine who featured periodically in *World Health* to help place contemporary doctors and scientists in a venerable tradition of defying disease.

As a relatively young agency, WHO required support for and confidence in its actions: it needed to project an assured view. It also needed to address the idea of glamour around chronic diseases which, it was said, dissuaded professionals. But as the above analysis has shown, the challenge of visually representing the issue meant that WHO fell back on a tried and tested valorisation of technology. The combined picture of chronic disease control was one of high adventure, but it was a story mainly about the medical establishment’s experience of and hopes about chronic disease rather than the individuals living with these conditions.

The overarching visual impression of chronic disease in this period, as put out by WHO, was also one lacking in emotion. Whereas for other diseases WHO pulled at the heartstrings, the clinical and calculating view of chronic diseases was largely about stopping emotion, namely worry and fear. It was also one in which the patient was marginalised: there was little sense of living with chronic conditions, just that they should be fixed like faculty machinery. Therefore, it presented a solution which did not address all of the issue. Some scholars have suggested that photography offers a powerful medium for conveying the truth about the experiences of those experiencing chronic diseases (Hassan 2016, 232). Yet, WHO’s early imagery was more akin to an aspirational vision. The staged and stylised photography was used not to record a story, but to project a hopeful vision that bio-medical interventions could offer possible solutions if an individual became ill.

### Finding the human angle

Such a conspicuous focus on science was not without its long-term problems. As Patterson (1989, x) outlines, in the 1970s the research emphasis of the cancer establishment began to be challenged by those insisting that the best ‘weapon’ was not high-tech research and therapy, but prevention. Greater criticism of the medical model of disease also arose, with a more social model of disabling conditions emerging in response (Oliver 2009, 41–57). This took place alongside recognition that conditions occurred in a social and cultural context and were affected by structural impacts and disadvantages which required more detailed understanding (Syme 1981). Furthermore, technology-driven, disease-focussed programmes had not delivered the anticipated successes. WHO’s Global Malaria Eradication Programme, for instance, had provided some benefits, but not the promised eradication. Moreover DDT, once hailed as a wonder weapon in the fight against malaria, had been shown to cause harm. In light of such realisations, WHO steadily began to switch from a ‘top-down’ approach to disease interventions to a ‘bottom-up’ one, emphasised in the movement for primary healthcare in the 1970s and characterised by community action for health and health education (Lee 2009, 72–74). Indeed, better information on the consequences of chronic conditions was seen as making an essential contribution to this goal, as part of a greater interest in environmental and lifestyle causes such as diet, nervous stress, pollution, heredity and individual susceptibility (Cantor 2007b, 23). A renewed focus on chronic diseases was amplified by the fact that they were no longer seen as just a problem for developed nations but as an issue facing countries around the world.

In 1970, John Higginson, Director of the International Agency for Research on Cancer, wrote in *World Health* of the growing public disillusionment which extended from expectations generated by the progress of molecular biology and chemotherapy in the 1950s and 1960s (World Health 1970, 39–44). Despite the fact that new research was steadily revealing more of the picture, people had become accustomed to the concept of breakthroughs. As WHO engaged with journalists and editors, it acknowledged that stories in the medical sections suggested that the media was interested only in dramatic and sensational events. This continued to be a concern for a long time: the problem throughout the latter decades of the 20th century was that medical advances, including organ transplants, gene-splicing and test-tube babies, continued to attract media focus, but contemporary public health priorities were different (WHO WPRO 1983, 12). Participants at a 1981 workshop to examine the relationship between the media and health recommended that global media outlets should resist focusing on sophisticated medical technology and infrastructure and instead emphasise the prevision of basic necessities (WHO Regional Office for the Western Pacific 1981, 125). Encouraging this shift, however, would not be easily or quickly achieved.

The 1970s saw the creation of a dedicated WHO Division of NCDs, again reflecting renewed interest and urgency. The surviving records of this Division illustrate the attention paid to the psychosocial factors acting in the prevention and control of chronic diseases, as well as the behaviours involved in seeking healthcare and adhering to treatment recommendations. It championed various community-based programmes for cardiovascular diseases, which began in Europe and the USA in the late 1960s and early 1970s and expanded to other NCDs mainly because of the common risk factors (Nissinen, Berrios, and Puska 2001, 963–970). Projects such as Monitoring Trends and Determinants in Cardiovascular Disease, a major international collaborative study into the determinants of cardiovascular disease established in 1979, and the Finnish North Karelia project to control cardiovascular diseases, demonstrated alternatives to pinning hope on technological revolutions.

This shift in focus fed through into the media image created and distributed by WHO. Of course, the imagery depicting high technology and medicalised settings was not immediately expunged: stories such as 1972’s ‘Heart Surgery: Coming of Age’ continued to celebrate innovations like synthetic aortic valves and implantable artificial hearts (World Health 1972). They continued to capitalise on the aforementioned appetite for innovations in treatment and detection. Yet, WHO now offered a range of different perspectives geared towards reshaping the narrative. The North Karelia project is a case in point. Following intensive health education focused on non-smoking, regular exercise and reduced intake of animal fats and salt, the region’s mortality from coronary heart disease dropped dramatically. The script for a WHO film spotlighting the project underscored the desired change in emphasis: it should be made clear that ‘modern technology could not produce good health in this population. It could only try to stave off the disastrous effects…The only ‘cure’ for these ailments was actually within the Finns themselves’ (Romer 1994).

From the early 1970s onwards, there is a discernible trend within *World Health*’s chronic disease photo stories towards content which focused on the effect on people’s lives rather than the progressive advancement of technology. Photo stories now encouraged viewers to feel for the people shown, and to understand their experiences and the challenges they faced, and use this as a basis to encourage them to reflect on their own health. In a 1970 special issue on cancer, for instance, readers were introduced to Jack Oelker, a dairy farmer from Ohio who had undergone surgery for a tumour of the jaw in the 1950s (World Health 1970). Fit and well since then, the article described how Oelker led an active life. In comparison with previous years, the accompanying photograph of Oelker, carrying a child on his shoulders, lacked any hint of medical context. It was foremost a picture of health, happiness and vitality, focused on human relationships and emotions. This was part of a growing tranche of photo stories which depicted chronic disease patients leading lives defined as normal, accomplishing everyday tasks and coping with challenges with assistance from friends and family.

No longer did *World Health* just focus on adults either. In a 1971 special issue on diabetes, readers were introduced to Jacqueline aged 10.5 years who, having been treated at a clinic since 1968, had learnt the basic principles of diet and how to inject with insulin (World Health 1971). In the accompanying photographs the person, as well as the life-saving insulin, was clear and presented as equally worthy of attention. In one shot, a reversal of the cobalt bomb image described earlier, Jacqueline peered down at the comparatively tiny syringe as she injected herself, suggesting medicine and technology being in the hands of humanity rather than vice versa. Such photographs showed doctors and patients in collaboration, the latter doing their duty by getting checked, following advice and generally adopting a responsible course with regard to their health. Jacqueline was also part of a generally more prevalent depiction of childhood. Director of the Division of NCDs, Dr Grabauskas wrote to colleagues of his conviction that ‘the roots of chronic diseases lie in childhood…preventative actions promoting healthy lifestyles early enough are most promising as a long-term strategy to combat the epidemic of chronic illness’ (Grabauskas 1986). Although not commonly associated with chronic diseases because of usually late onset, children could also be used to inspire emotional responses to these conditions and get parents to reflect on their own mortality. Well-represented elsewhere in WHO’s visual arsenal as a way to incite pity, here children were used to speak of hope.

**Figure 3:**
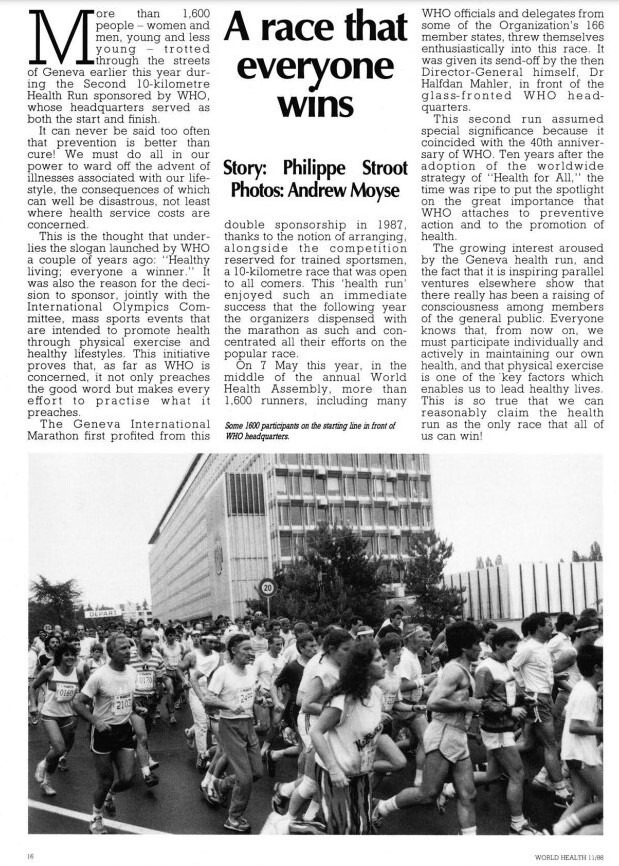
A race that everyone wins. Bland J., Viedma C. and Chrétien J-F. 1988. “World Health: the magazine of the World Health Organization: November 1988 (full issue): my health first. World Health, (November), 2–31”. World Health Organization (page 18). Copyright WHO 1988. Figure permission obtained. https://iris.who.int/handle/10665/311175

Photographic narratives also shifted to emphasise community action. While talks, lectures and group meetings constituted one way of picturing pulling together and staying healthy for one another, the chief way WHO did this in relation to chronic conditions was through physical exercise and team spirit (figure 3). This also made a connected point about prevention: the message chosen for 1986’s World Health Day was ‘Healthy Living: Everyone a Winner’, which heightened the emphasis on the positive actions that individuals and communities could take to promote health. The corresponding *World Health* special issue carried a stylised illustration of a footrace on the front cover, and inside photographs focused on team sports almost exclusively, including soccer, rowing competitions, fun-runs, hockey as well as tug-of-war and wrestling. As such, the issue boldly equated a healthy lifestyle with victory through sport, and communities with teams. Sporting heroes and other inspirational individuals also appeared more frequently: as figure 3 describes, WHO had recently fostered a collaboration with the International Olympic Committee. Another feature, in 1971, on William Talbert, former captain of the US Davis Cup team, was illustrated with Talbert playing tennis to underscore his message that: “I did not want to be considered ‘different’ from my fellow players…I didn’t like the term ‘fellow sufferers’. I didn’t consider myself a sufferer of anything” (World Health 1971). While it represented a change in tone when compared with chronic diseases being conquered with war-like means, the overarching narrative was one in which people could still ‘win’ or ‘lose’. The victory narrative remained dominant, even though it took on a different form.

While useful on the one hand, such a focus had the potential to create a popular mindset which believes that such problems can be solved by exercise alone and that people who suffer are therefore lazy, or have neglected their body. This accords with another of the narratives described by Frank, that of the quest. Quest stories ‘meet suffering head on; they accept illness and seek to use it’ (Frank 2013, 115). However, the risk of quest narratives, he argues, is that they can present the process as too clean and the transformation as too complete, and they can implicitly deprecate those who fail to rise out of their own ashes (Frank 2013, 135). Indeed, in the latter part of the 20th century, WHO’s photojournalism also moved to consider more prominently villain-victims such as smokers, drinkers, drug users, in which the person became their behaviour. 1970’s cardiovascular disease special issue featured an outlandish photograph of a significantly oversized man smoking a pipe. His photograph took up the full double page, with the viewer’s gaze centred on his substantial waistline. The accompanying caption read simply ‘obesity favours to hypertension’ (World Health 1970). Another article made the sober point that it was desirable for a patient with hypertension to adopt a diet which would help lower cholesterol levels, but this was illustrated with another full-page photograph of a middle-aged female seated at a table laden with desserts, in the act of putting a spoon to her open mouth (World Health 1978).

With regard to alcohol consumption, the aforementioned ‘Healthy Living Everyone a Winner’ special issue showed an elderly woman seated at a bar filled with empty glasses. Physically, she looked pale and drawn; resting her head on one hand she gazed dejectedly at apparently nothing in particular. The viewer was therefore invited to consider her thoughts, a reading supported by the caption which described ‘Alcohol-related problems: the disruption of family life, neglect, violence and traffic accidents’ (World Health 1986). To further emphasise the destructive power of alcohol the overarching story was titled ‘Towards an Alcoholic Holocaust?’ The same issue highlighted ‘famous victims’ Orson Welles and Yul Brinner whose renown did not ‘confer immunity from the “diseases of affluence”’. Finally, a special issue on diabetes included an intriguing reportage-style photograph of two men in an urban setting seated on the floor. One swigged voraciously from a bottle, while the other confronted the photographer, hand outstretched to push them away. The caption read ‘Alcohol—false and fatal friend’ (World Health 1991). It is possible that this was staged, but by the look of real anger on the man’s face and the blurred hand in the foreground it appears to have been captured authentically, and in all likelihood was meant to be interpreted as such. While this was certainly not the routine practice of WHO photographers this suggests a degree of intrusion to get the required photo, the apparent lack of consent made acceptable because these were marked out as apparently ‘faulty’ people.

As the double disease burden facing countries around the world was recognised, *World Health* also moved to picture the chronic disease problem as an important concern for countries beyond Europe and the USA. Indeed, WHO proclaimed its wish to forge international cooperation and understanding, and stress a ‘common language’ of chronic disease (World Health 1975). Some photo stories sought to address entrenched assumptions linked to the idea that chronic conditions were associated with affluence, pointing out that ‘Africans Have Diabetes Too…’ (World Health 1971), and that there was of course a need for ‘Tropical Heart Research’ (World Health 1969). Such stories reported on different country-based programmes, setting the scene and providing visual evidence that these were common problems affecting people in all countries as well as displaying the efforts deployed in response. But many photo stories lacked meaningful imagery, or imagery that supported this new narrative. A photograph accompanying a feature on ‘The African Experience’ of cardiovascular disease depicted women sitting in a circle with young children in a typically rustic village setting. The accompanying caption however noted that ‘In isolated areas of Africa, blood pressure does not rise with age; one line of speculation attributes this to the absence of stress. But an adequate explanation has yet to be found’ (World Health 1978). Here, both photograph and text arguably worked to reinforce longstanding assumptions and stereotypes about African ways of life, rather than counter them. Community activities and technological solutions all featured in a varied photographic tapestry, which further suggests a degree of difficulty in getting the ‘right’ look. Again, the focus on technology was valued, but in light of the move to primary healthcare, WHO wanted to champion other approaches. However, this in itself proved problematic: there is evidence that communities in some countries were bemused, sometimes angry, that they were being offered and promoted community programmes and basic services rather than the advanced solutions which had been so ardently showcased for decades and which were assumed to be superior (Medcalf and Nunes 2018, 420–21). This suggests on the one hand the success of the technological focus, and the failure of subsequent narratives to supplant it.

Beyond such hints, it is difficult to determine the precise effects of this imagery on those who saw it, be this in *World Health* or reproduced in a third-party publication. Yet as the deadline for ‘Health for All’ approached, WHO appeared to be pleased with its efforts. A progress report prepared for the 1992 World Health Assembly described consciousness-raising campaigns made during the 1980s and the dramatic fall in deaths from ischaemic heart disease between 1970 and 1980, which it put down to a positive combination of legislation and education. The report also noted the intensification of media coverage on subjects such as lifestyles, the environment, cancer, smoking and drug abuse. WHO reiterated the priority of generating and disseminating timely, pertinent and up-to-date information to decision-makers, the public and to health and development staff. But it also recognised limitations such as the need for a favourable social climate and an adequate support system to make it feasible for people to transform ideas into action (WHO 1992). The curtailment of *World Health* in 1998 marked the end of this particular form of messaging, although photography continued to play a key role in chronic disease campaigns and exhibitions.

## Conclusion

This article has sought to address the imbalance in coverage between the representation of communicable diseases and chronic conditions. There is still much to be done in this regard, and while WHO was not the only organisation releasing such content, its significant role and reach make it an ideal starting point. We can see that as the aetiology of chronic conditions was still being explored, WHO followed a trusted method of representing them as ‘enemies’ that were intangible, amorphous and sometimes difficult for lay people to understand fully. They were less united by their risk factors as much as how they could be tackled, predominantly by technology. The emphasis was on chronic conditions as something to be cured or palliated rather than prevented despite growing evidence within and outside the organisation. The imagery was not challenging or controversial, but fitted into set organisational beliefs and goals. Over time, WHO’s media teams developed this narrative to bring in a human angle, telling stories about lives affected by chronic disease. Yet as in the case of the shift to primary healthcare, ‘community’ proved challenging to define visually, and the visual vocabulary also turned to a more vociferous blaming of faulty habits.

WHO representations found it difficult to transverse the idea of the ‘sufferer’, often evoking contradictory (and circular reinforcing) responses. That chronic conditions could (or should) be avoided is initially juxtaposed against the potential of scientific medicine to palliate consequences. Photographs also struggled to capture the more social determinants of health because of their focus on personal experiences or individual stories, consistent with restitution or quest narratives. The focus was on biomedical/technological and individual lifestyle rather than an engagement with more social understandings. Capturing these social conditions is, however, more complex and abstract, in which much is expected of the reader to make the connection. This perhaps explains the interest with individuals and their stories: the impact is immediate, relatable and human (Riessman 2007).

Understanding these contractions enables us to establish the potential value of photography in raising awareness, and identify the potential moral hazards created by its use. While many have emphasised the particular potential of photography in representing illness, and bringing to the fore issues which have been neglected or which suffer misconceptions, we also need to be aware of its limits in this regard. It can serve to make the invisible ‘visible’, but in doing so it generally works within existing frameworks of representation and visual vocabularies. As this article has shown, it is difficult to depart from conventions, and when this is achieved it risks losing its power or relevance. To paraphrase Susan Sontag, such photographs are the antidote and the disease (Sontag 1991, 179). The antidote in the sense that hope is pinned on their ability to change public perceptions, but a disease because in constantly reshaping and redefining issues they create the cultures which ultimately require a response.

This article has not sought to say what was ‘good’ or ‘bad’ representation, but to consider what this representation did in terms of the overarching picture it created. We have shown how the images were connected to the contextual and normative understanding of the time, including an initial emphasis on the promise of cure and confidence in healthcare professionals, as well as attempts to represent agency, while highlighting the dangers of risky behaviours. These challenges persist and offer a reminder that health remains a political concern, continually co-produced and cultivated through photographic images. While we have focused on WHO’s awareness campaigns, further study is needed into how other areas of the media present the chronic question. For instance, Dumit raises the question of pharmaceutical companies’ involvement in shifting the narrative as chronic conditions became an increasingly attractive market than acute treatments (Dumit 2012, 5–7).

How can future images reproduce and engage with these chronic illnesses? The ethical challenge facing photographic representation is to acknowledge the precarity and undesirability of chronic illness, while reflecting the active agency of those with chronic conditions, including the potential for stigma, when presenting their experience. Perhaps more importantly, however, future images will be expected to capture active engagement with illness, in a way that demonstrates how individuals can, through active agency, live well and flourish (Willen *et al*. 2022). However, as Roberts argues, ‘for individuals to flourish, they must be situated in societies that promote their flourishing’ (Roberts 2019, 201). Photographic images will have an important contribution in ensuring the context which empowers this flourishing. But ultimately this must be done with a recognition that picturing chronic conditions should not just be about creating awareness or finding something that will play well in the media. Careful thought needs to be given to the impact and long-term legacy of picturing practices.

## Data Availability

Data sharing not applicable as no datasets generated and/or analysed for this study.

## Footnotes

https://thepolyphony.org/2021/05/18/capturing-chronic-illness-bearing-witness-through-photography/
